# Prospects of Next-Generation Vaccines for Bluetongue

**DOI:** 10.3389/fvets.2019.00407

**Published:** 2019-11-21

**Authors:** Piet A. van Rijn

**Affiliations:** ^1^Department of Virology, Wageningen Bioveterinary Research, Lelystad, Netherlands; ^2^Department of Biochemistry, Centre for Human Metabolomics, North-West University, Potchefstroom, South Africa

**Keywords:** bluetongue, safety, efficacy, affordability, DIVA, acceptance, marketed vaccine, experimental vaccine

## Abstract

Bluetongue (BT) is a haemorrhagic disease of wild and domestic ruminants with a huge economic worldwide impact on livestock. The disease is caused by BT-virus transmitted by *Culicoides* biting midges and disease control without vaccination is hardly possible. Vaccination is the most feasible and cost-effective way to minimize economic losses. Marketed BT vaccines are successfully used in different parts of the world. Inactivated BT vaccines are efficacious and safe but relatively expensive, whereas live-attenuated vaccines are efficacious and cheap but are unsafe because of under-attenuation, onward spread, reversion to virulence, and reassortment events. Both manufactured BT vaccines do not enable differentiating infected from vaccinated animals (DIVA) and protection is limited to the respective serotype. The ideal BT vaccine is a licensed, affordable, completely safe DIVA vaccine, that induces quick, lifelong, broad protection in all susceptible ruminant species. Promising vaccine candidates show improvement for one or more of these main vaccine standards. BTV protein vaccines and viral vector vaccines have DIVA potential depending on the selected BTV antigens, but are less effective and likely more costly per protected animal than current vaccines. Several vaccine platforms based on replicating BTV are applied for many serotypes by exchange of serotype dominant outer shell proteins. These platforms based on one BTV backbone result in attenuation or abortive virus replication and prevent disease by and spread of vaccine virus as well as reversion to virulence. These replicating BT vaccines induce humoral and T-cell mediated immune responses to all viral proteins except to one, which could enable DIVA tests. Most of these replicating vaccines can be produced similarly as currently marketed BT vaccines. All replicating vaccine platforms developed by reverse genetics are classified as genetic modified organisms. This implies extensive and expensive safety trails in target ruminant species, and acceptance by the community could be hindered. Nonetheless, several experimental BT vaccines show very promising improvements and could compete with marketed vaccines regarding their vaccine profile, but none of these next generation BT vaccines have been licensed yet.

## Introduction

### Bluetongue Disease

Bluetongue (BT) is a hemorrhagic disease of wild and domestic ruminants caused by bluetongue virus (BTV) (1, 2). BT is one of the main veterinary diseases worldwide causing significant economic losses (3, 4). The outcome of BTV infection varies and depends on the pathogenicity of the virus strain and the susceptibility of the ruminant host. Indigenous ruminants in BT endemic areas, goats, and cattle are less susceptible than many sheep breeds from BT-free areas (5). Sheep can induce severe clinical disease (6, 7), whereas cattle rarely show clinical disease but are readily infected and are an epidemiologically important BTV reservoir. BTV is not contagious but transmitted by biting competent *Culicoides* midges (8), whereas several recently discovered BTV serotypes spread without midges by direct contact transmission (9–11). Virulent BTV can also spread oro-nasally or vertically (12, 13) and have been reported in the field (14–16). A role of transplacental transmission in overwintering has been hypothesized (17), and trade of pregnant heifers can transport infectious BTV over long distances potentially causing outbreaks in former BT-free areas by delivery of viremic fetuses (18).

### Bluetongue Virus

**Figure 1:**
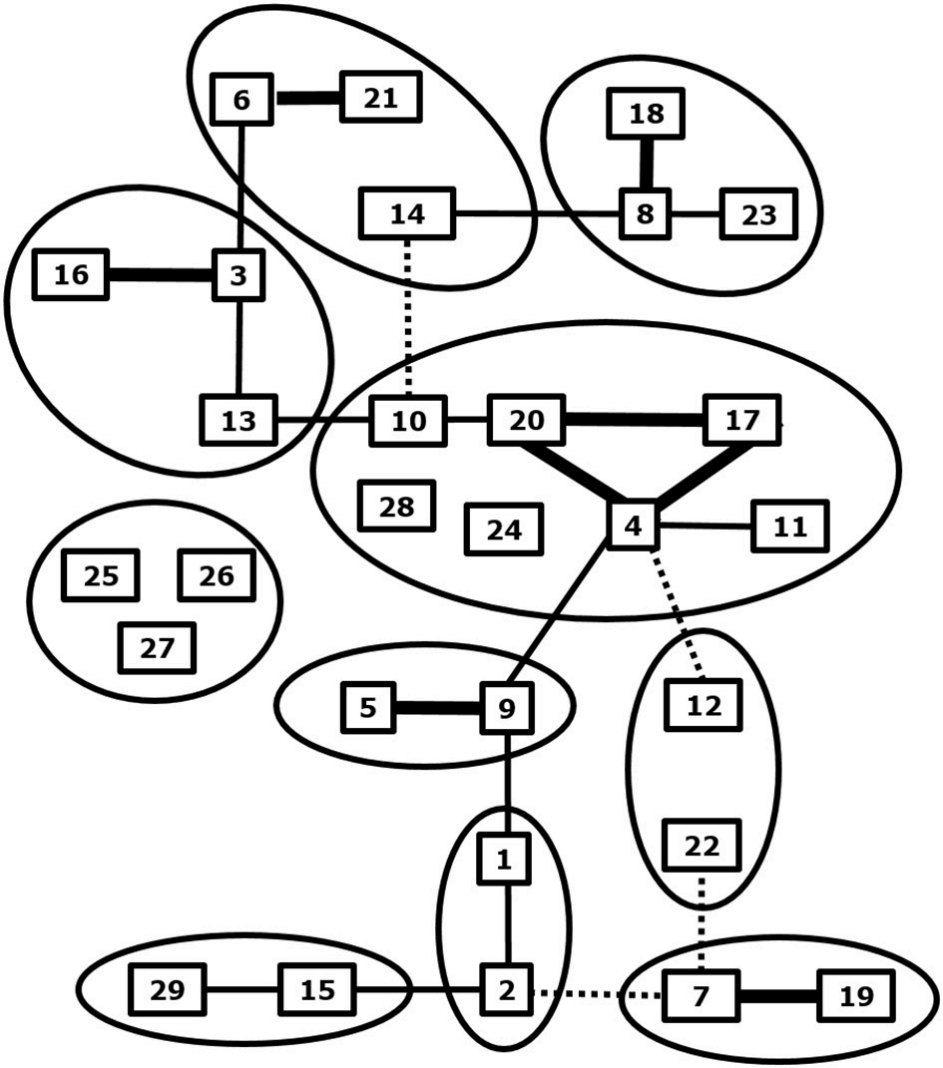
Phylogenetic and neutralization relationship between BTV serotypes. Related BTV serotypes based on genome segment 2 expressing serotype specific immunodominant outer shell protein VP2 are grouped by circles. Cross neutralization between BTV serotypes is indicated by lines; strong (thick), some (normal) and weak neutralization (dashed). Adapted from Erasmus et al. (28), and Maan et al. (30) and updated for BTV27-29 from Bumbarov et al. (31), Wright (32), and Zientara (33).

BTV is the prototype orbivirus within the genus *Orbivirus* of the family of *Reoviridae* (19). Orbiviruses are non-enveloped viruses and consist of a three-layered icosahedral capsid containing a segmented genome. Ten double stranded RNA genome segments S1-10 encode seven structural proteins VP1-7 and at least 4 non-structural proteins NS1-4 (19–22). BTV infection results in a transcriptionally active core particle producing mRNAs of all ten segments which are released into the cytoplasm (23). BTV was recovered from core-derived mRNAs about 20 year later (24), and BTV was rescued by double transfection of ten synthetic RNA run-off transcripts from cDNAs, which is known as reverse genetics (25). Reverse genetics has opened endless possibilities to study viral functions in the BTV infected cell, in particular of non-structural proteins (26, 27). The BTV species or serogroup consists of many neutralization groups hardly showing cross-neutralizing antibodies and poor cross-protection (28, 29) (Figure 1).

### BTV Serotypes

BTV serotypes 1–24 have been recognized by cross neutralization assays and have been confirmed by phylogenetic analysis of S2, which encodes the serotype specific and immunodominant VP2 protein of the outer shell (34) (Figure 1). Eastern and western topotypes of many serotypes are recognized, suggesting segregation a long time ago (35). In the last decade, at least five new BTV serotypes have been discovered (30, 32, 33, 36–38) (Figure 1). BTV25-27 are known as “atypical BTV,” because, in contrast to typical BTV1-24, these are exclusively found in small ruminants, are not pathogenic, spread by direct contact transmission, and cannot be cultured in *Culicoides* cells (9, 10, 39–41). BTV28 is also transmitted by in-contact transmission but causes clinical disease and its VP2 is closest related to the main BTV genotype group consisting of serotypes 4, 10, 11, 17, 20, and 24 (31). BTV29 has been isolated from Alpaca in South Africa and is closely related to serotype15 based on phylogenetic and cross neutralization analysis (32). Two recently found BTVs are not studied in detail yet, but are proposed as new serotypes according to phylogenetic analysis of S2 sequences (37, 38). Discovery of more typical and atypical BTV serotypes in livestock and wild ruminant species can be expected by intensified surveys with more sensitive and new technologies (42). These “to-be-discovered” BTVs are likely not pathogenic but could become of concern, since mutations and reassortment with virulent BTV serotypes quickly change virus characteristics, including pathogenicity and the epidemiology.

### Epidemiology

For a long time, BT has been widespread in tropical and subtropical regions all over the world, but is restricted and dependent on the local presence of specific competent biting midges in different parts of the world (43). At the end of the twentieth century, BT-affected areas had started to expand to former BT-free areas with a moderate climate, and outbreaks caused by emerging BTV serotypes have been frequently reported since then (44, 45).

BTV1, 2, 4, 9, and 16 entered southern Europe associated with expansion of the *Culicoides* imicola vector from northern Africa. In 2006, BTV8 (BTV8/net06) emerged in north-western Europe (15, 46), and was spread by indigenous midge species of the *Culicoides* obsoletus complex (47–49). Likely, global warming favors expansion of well-known competent midges species as well as increases the vector competence of some midge species. Subsequently, BTV8/net06 survived the inter-seasonal “vector-free” period known as “overwintering” and was spread to many European countries resulting in the largest recorded BT outbreak. Vaccination campaigns eradicated BTV8 in most European countries but BTV8 re-emerged in France in 2015 after 5 years of silence (50). This virus variant, BTV8/fr15, caused a lower viremia, less severe disease and virus transmission was much slower suggesting a lower vector competence. Likely, one or more amino acid changes in BTV8/fr15 are involved in this changed phenotype (51). In 2014, a new BTV reassortant of serotype 4 emerged in south-eastern Europe (52), and expanded to a wide area into Italy and mainland France in following years. Like BTV9 in this area, this BTV reassortant is likely spread by *C*. obsoletus, since *C*. imicola has not been found in the Balkan region. In 2017-18, BTV3 “jumped” from Tunisia to the Italian islands Sicily and Sardinia (53–55).

Many serotypes are endemic in Northern Australia but BTV5 emerged in 2015 for the first time (56). Additionally, the Australian authorities have moved the installed border of the BT-free area, including quarantine centers, southwards due to expansion of the BTV affected area (https://animalhealthaustralia.com.au). In large parts of the USA, serotypes 2, 10, 11, 13, and 17 are endemic and temporarily expand further northwards up to Canada depending on annual environmental conditions (57). In addition, 11 serotypes circulate in south-eastern USA, mainly in Florida, and reassortants of serotype 3 have been recently isolated in several states in the USA (58). Many serotypes are endemic in large parts of South America (59), but little is known of the BT-history on this continent.

BTV constantly evolves by mutations and reassortment events leading to invasion of new variants in areas with susceptible hosts and competent midges (60, 61). Additionally, global warming and climate change likely contribute to expansion of BT affected areas (57, 62). Intensified movements of animals and animal products will also increase the chance on incursions of BT. In conclusion, (re-)emerging BT outbreaks can be expected all over the world. Preparedness on this threatening situation should be of high priority to safeguard the health and production of ruminant livestock in developing and developed countries (63).

### Control of Bluetongue

BT control by restrictions on trade and movements and vector control is inadequate, insufficient, non-proportional, and expensive compared to the impact, while destruction of infected ruminants is not acceptable by the community. The failure in disease control is mainly caused by uncontrolled spread of BTV by infected midges. Vaccination is the preferred method for BT control (64–66). Prophylactic and emergency vaccination have contributed to BT control and significantly reduce economic losses caused by mortality, morbidity, reproduction problems, animal losses and lower milk production (67–69). The success of vaccination campaigns is best demonstrated by the eradication of BT in many European countries after the devastating outbreak caused by BTV8. Eradication of BT strongly depends on participation of animal owners to reach a high vaccination coverage of livestock, the used vaccine, and the field situation, like the presence of wildlife species as BTV reservoir and thus potential re-incursions (70). Still, intensified and repeated monitoring for several years is required to proof the absence of BTV circulation. Serological monitoring in the vaccination population is hindered by lack of specific assays to discriminate between infected and vaccinated animals, but is feasible by testing of non-vaccination sentinel herds or testing of selected new-born (non-vaccinated) animals after maternal antibodies have been disappeared.

## Vaccines

### Vaccine Profile

**Figure 2:**
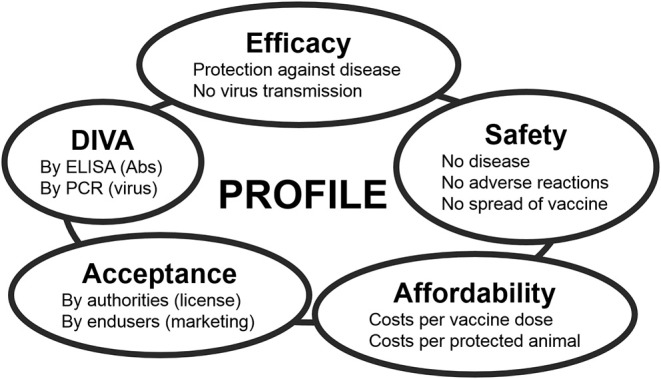
The main standards for modern veterinary vaccines. Each standard can be subdivided into several criteria. The ideal vaccine completely meets all these criteria, but profiles of marketed and experimental vaccines mostly compromise between standards depending on the foreseen aim of vaccination and on the field situation Feenstra and van Rijn (70).

The ideal BT vaccine is efficacious, safe, affordable, and has been licensed. Preferably, the vaccine is a DIVA vaccine [Differentiation Infected from VAccinated individuals (71)] to support eradication and to safely allow trade and movement of DIVA-vaccinated and BT-naïve animals. Each of these main standards for vaccines is the sum of several criteria (Figure 2). Efficacy is divided into protection against disease and blocking of onward virus transmission. Further, protection should be quick and lasting, preferably lifelong. Because of many neutralization groups, the ideal vaccine is broad protective or is tailor-made to anticipate on circulation of multiple serotypes. Safety is subdivided into non-pathogenic and no adverse effects in ruminants of different status, like pregnant and young animals. Further, the vaccine should not spread into the environment, like through uptake and spread by midges or in-contact transmission. Affordability consists of costs/dose and price/protected animal. The costs/dose depends on development costs and production costs, while the price/protected animal also depends on vaccine efficacy and vaccination strategy, like one single vaccination or repeated vaccinations to achieve lasting protection. Consequently, affordability is also associated with the value and lifespan of the susceptible species in a certain country or region. DIVA is subdivided into genetic DIVA to detect acute BTV infections, and serological DIVA to massively monitor (vaccinated) ruminant populations for anti-BTV Abs in order to detect past BTV circulation. Finally, for massive use and success of vaccination campaigns, the ideal BT vaccine should be licensed, and of course, its own success will increase the acceptance by users.

### Marketed Vaccines

**Figure 3:**
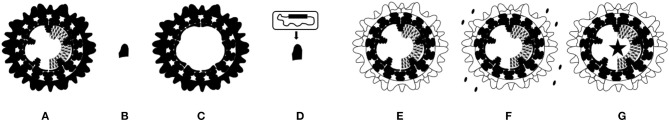
Schematic representation of marketed and experimental BT vaccines. **(A)** Marketed vaccine is based on entire live-attenuated or inactivated BTV. **(B)** Subunit vaccine is based on BTV protein(s) produced in artificial systems, and mostly contains the here presented serotype specific outer shell VP2 protein. **(C)** VLP vaccine consists of empty virus particles produced in artificial systems consisting of BTV proteins VP2, 3, 5 and 7. **(D)** Viral vector vaccine is nonBT-virus expressing one or more BTV proteins. Here, a VP2 expressing viral vector vaccine is presented. **(E–G)** The exchanged serotype specific outer shell proteins VP2 and VP5 are indicated (white). **(E)** “Serotyped” LAV and inactivated BT vaccine are based on a common LAV or a production BTV backbone, respectively. **(F)** DISC vaccine lacks expression of an essential BTV protein, and must be produced by *in trans* complementation as indicated. **(G)** DISA vaccine and NS4 knockout vaccine lacks expression of nonessential NS3/NS3a or NS4, respectively (asterisk).

Currently, two types of marketed BT vaccines are used in large parts of the world, conventionally live-attenuated vaccines (LAVs) and inactivated BT vaccines. Both are based on whole BT-virus, and induce immune responses against immunogenic BTV proteins (Figure 3A).

#### Live-Attenuated Vaccines

Protection by LAV is serotype specific, although some cross neutralization has been noticed (28) (Figure 1). A cocktail containing LAVs of 14 serotypes did not result in broad protection of sheep (72). However, multi-serotype LAV cocktails can induce neutralizing antibodies against not-included serotypes, and subsequent vaccinations with three different pentavalent LAV cocktails induce broad protection (73). These pentavalent cocktails contain 15 different serotypes in total; bottle A (serotypes 1, 4, 6, 12, and 14), bottle B (serotypes 3, 8, 9, 10, and 11), and bottle C (serotypes 2, 5, 7, 13, and 19). These LAVs induce some clinical reactions commonly including a transient febrile reaction [reviews; (72, 73)], and may cause teratogenic effects; abortions, stillbirths, fetal malformations, temporary infertility in rams, and ewes, and reduced milk production [(74) and included references]. Further, these LAV cocktails require a correct order of use, since bottles B and C contain under-attenuated LAVs, which could lead to a higher incidence of disease if used as prime vaccination. Adverse effects have been shown after vaccination with LAVs of serotypes 2, 4, 9, and 16 in the Middle East and after temporarily use in southern Europe [reviewed in (75)]. More importantly, LAV viremia is sufficiently high for uptake by midges and thus onward spread, and these LAVs are no longer used in South Europe (76–79). Nonetheless, vaccination with LAVs prevent severe clinical disease and reduce viremia of wild type BTV (wtBTV) (80). Since the exact mutations and attenuation sites in LAVs are unknown and are likely located on different genome segments for each LAV, reversion to virulence and virulent variants by reassortment are possible (75, 81, 82). Despite of the debatable safety of conventionally live attenuated vaccines, these are used in several parts of the world, since LAVs are cheap and effective, while adverse reactions are marginal in local breeds (83, 84).

#### Inactivated Vaccines

In the 1970s and 1980s, inactivated BT vaccines have been developed in the USA but have not been licensed (85–88). The emergence of several BTV serotypes in Europe re-activated this approach. Inactivated BT vaccines for some serotypes have been licensed in Europe and are produced at industrial level on request in case of emergency [reviewed in (89)]. Inactivated vaccine cross protects early after vaccination by innate immunity but protection switches to serotype specific protection later on (90). In general, protection by inactivated vaccine is serotype specific, although some heterologous protection against other serotypes can be induced but hard to predict (91), whereas inactivated BT vaccines for serotypes 1 and 4 showed negative interference for serotype 4 (92). Inactivated BT vaccines are completely safe and, although for a limited number of serotypes, the only type of BT vaccine currently registered in Europe. Success of inactivated BT vaccines is the best demonstrated by eradication of serotypes 1, 2, 4, and 8 in several European countries after massive vaccination (75, 93–95). Field application shows very good records and neutralizing antibodies persist for many years (96–98). Details of BTV antigen production, formulation and adjuvant have not been published in detail. The amount of antigen per dose of inactivated BT vaccine typically corresponds to approximately 10^7^ TCID_50_ virus (99), which is about 100 times more than 10^5^ TCID_50_ virus/dose for LAVs. Inactivated BT vaccine is therefore more expensive but safer than LAV. Particularly, inactivated vaccine is more expensive for large ruminants, since booster vaccination is recommended (100). Inactivated BT vaccines are potential DIVA vaccine, since non-structural (NS) proteins can be removed from produced BTV antigen. ELISAs to detect antibodies directed to NS proteins have been published (101–103). However, stringent purification to remove NS proteins from crude extract of produced BTV particles is required and will increase the production costs of inactivated BT vaccine.

In conclusion, LAVs and inactivated BT vaccines are available, although for the latter only for a limited number of serotypes. Despite of several success stories in different parts of the world for these marketed vaccines, both vaccine types have their specific shortcomings. The current choice of vaccine depends on many aspects, including the objective, local legislation, and their vaccine profile taking pros and cons into account. Clearly, there is ample room for improvement of currently used vaccines (70).

### Promising Experimental Vaccines

Several experimental BT vaccines are under development, and are divided into; (1) vaccines based on BTV proteins, e.g., VP2 subunit and virus like particles (VLPs); (2) viral vector vaccines based on nonBT-virus expressing one or more BTV proteins, and; (3) vaccine platforms based on BTV (Figure 3). These approaches are subject of vaccine research for many years and show improvements compared to marketed vaccines.

#### BTV Protein Vaccines

Experimental protein based BT vaccines all include the serotype specific immunodominant VP2 protein (Figure 3B). Protein production has been studied in bacteria (104), in insect cells (105–108), in yeast (109), and in plants (110–112).

##### VP2 Subunit Vaccines

A protective dose by VP2 could be reduced 50% by adding VP5 protein, but adding of Freund's adjuvant or other BTV proteins did not further enhance the protective immunity (113). Recently, 150 μg purified VP2, NS1, and NS2 proteins with the immunostimulating complex AbISCO-300 showed a good cellular and humoral immunity in cattle (114). This candidate protects calves 3 weeks after booster vaccination. T-lymphocytes were mainly raised against NS1 and are cross reactive amongst different serotypes because of a higher conservation of NS1 protein. This suggests that the cellular responses to NS1, and likely NS2, can be the fundament of vaccine for other serotypes by varying VP2 protein (115). Other experimental subunit vaccine candidates have been developed and showed promising results but are mostly not tested in the natural ruminant host yet (110). Two domains of VP2 (aa 63–471 and 555–956) and VP5 lacking the first 100 amino acids are produced in bacteria as soluble fusion-proteins with glutathione S-transferase (116). Immunized IFNAR^(−/−)^ mice expressed neutralizing antibodies and survived homologous challenge without clinical signs after booster vaccination with 15 μg of the VP2 domains and 25 μg VP5. Addition of VP5 protein enhanced the immunity but addition of VP7 did not. VP2, VP7, and NS1 were incorporated in MuNS microspheres (117). An advantage of these inclusions using the baculovirus expression system is the easy method of purification and their potent adjuvant activity (118). IFNAR^(−/−)^ mice immunized with these particles without adjuvant induced both humoral and cellular immune responses, and these mice were protected against lethal BTV challenge. VP2 has also been fused to the antigen presenting cell homing (APCH) molecule, and was produced in insect cells (119). APCH fusion has been demonstrated to improve the immune responses induced against many different antigens. This antigen formulated with oil adjuvant Montanide ISA50 showed a good humoral immune response in cattle with a minimal dose of 900 ng, but a BTV challenge has not been performed. IFNAR^(−/−)^ mice have also been vaccinated and specific CD4+ and CD8+ T cells producing IFNγ following virus stimulation were observed, whereas lower levels were recorded for mice immunized with only VP2. Part of the VP2 gene has also been expressed using *Pichia pastoris* (109). High level of secreted expression was achieved, and the produced protein is immunogenic in rabbits.

##### VLP Vaccines

VLPs are empty virus particles consisting of structural proteins and are investigated as vaccine candidates for decades. BTV VLPs consist of VP3, VP7, VP2, and VP5 which are expressed in insect cells using baculovirus expression (120–124), and by the *Nicotiana benthamiana* plant and the cowpea mosaic virus based HyperTrans plant transient expression vector system (125) (Figure 3C). A cocktail of VLPs for several serotypes 1, 2, 10, 13, and 17 protected against all five serotypes and partially protected against some other serotypes (126). Huge sheep trials with 50–200 sheep per trial showed afforded protection by VLP vaccination against homologous challenge (127). Despite of all these efforts and promising results, VLPs have not been manufactured in that time. Most likely, marketed inactivated BT vaccines are much cheaper to produce, and equally safe. Protein and VLP production in plants have become an increasingly popular alternative for artificial protein production of complex high-value proteins, and might become cost effective.

##### New Inactivated BT Vaccines

A vaccine platform for production of inactivated BTVs has been developed (128). Reverse genetics for BTV1 (25) was used to exchange serotype specific outer shell proteins of 18 BTV serotypes (Figure 3E). The prototype “serotyped” inactivated BT vaccine for serotype 8 induces serotype specific neutralizing antibodies and protects sheep against virulent BTV8 challenge. This synthetic biology approach will optimize production and will shorten the time to produce inactivated BT vaccines for new and emerging serotypes.

Summarizing, protein based BT vaccines provide opportunities compared to commercial inactivated BT vaccines. VP2 subunit and VLP vaccines contain specific BTV proteins and are produced in artificial production systems. Therefore, these require minimal biocontainment facilities and can be DIVA compliant. In particular, guaranteed vaccine safety by lack of infectious BTV or contamination of animal related viruses is a great advantage of protein based BT vaccines.

#### Viral Vector Vaccines

Different viruses have been explored as vector for the development viral vector vaccines expressing one or more BTV proteins intracellularly and therefore inducing cytotoxic T lymphocyte responses in addition to humoral responses (Figure 3D). Replication of viral vector vaccines is abortive, and will not induce clinical signs associated to BTV infection. Canarypox virus expressing both VP2 and VP5 induced sterile immunity in sheep (129), whereas capripox viruses expressing VP2, VP7, NS1, or NS3 induced partial protection (130). Myxomavirus expressing VP2 or both VP2 and VP5 also partially protects sheep against BT (131). Bovine herpes virus expressing VP2 targeted to the cell membrane also induced partial protection in IFNAR^(−/−)^ mice (132). Immunization of IFNAR^(−/−)^ mice with equine herpes virus expressing both VP2 and VP5 protects against mortality but mild clinical signs were observed after challenge (133). All these viral vector vaccine candidates require booster vaccination, and most of these did not completely protect mice or the ruminant host. A promising exception with regard to previous research on viral vector vaccines is the wide immunoprotection of IFNAR^(−/−)^ mice by inoculation with modified vaccinia Ankara virus (MVA) vector expressing an immunodominant epitope on BTV-NS1 protein (134). Research in ruminants is needed to study broad and effective protection in the target species.

The main obstacle of viral vector vaccines is immunity against vector associated antigens by previous exposure [reviewed in (135)]. Priming by DNA vaccination followed by vaccination with viral vector vaccine can partially overcome the disadvantage, and DNA vaccine is a potent inducer of Th1 responses. However, reliability and effectiveness of DNA vaccines are questionable by inefficient delivery and is therefore still limited [reviewed in (136)]. Prime vaccination with BTV1 pCAGGS DNA vaccine (137), followed by recombinant fowlpox virus vaccine for VP2, VP5, or both proteins induced T-cell response in BALB/c mice, and high titres of neutralizing antibodies in both mice and sheep but protection against BTV was not investigated (138). Similar strategies showed protection in IFNAR^(−/−)^ mice with plasmids encoding VP2, VP5, and VP7 and MVA vector (139), and with NS1 instead of VP5 showed a higher T-cell response and heterologous immunity (140). VP2 expression induced protection to homologous challenge similar as expression of VP2, VP5, and VP7 together, which indicates the importance of serotype specific immunodominant VP2 protein (141). The prime-boost strategy with DNA and viral vector vaccines is promising but more research in the susceptible ruminant host is needed.

Viral vector vaccines are potential DIVA vaccines and safety with regard to lack of infectious BTV is guaranteed. In addition, production of viral vector vaccines requires a permitted (lower) biocontainment level and will lower the production costs. Some viral vectors have been registered and likely further reduces the costs to license these viral vector vaccines for BT.

#### Replicating BT Vaccines (MLVs)

Development of reverse genetics for orbivirus prototype BTV was a breakthrough in orbivirus research (25), and has been optimized to robustly generate BTV mutants, modified-live vaccines (MLVs) and “synthetic” reassortants (142–144). Reverse genetics has been used for fundamental and applied research to investigate viral functions in the BTV infected cell. Synthetically derived BTV is indistinguishable from its virulent or nonvirulent ancestor BTV (145). The segmented BTV genome is very flexible, and many desired so-named “synthetic” BTV reassortants can be generated easily using a set of 10 selected RNA run-off transcripts (145, 146). One example as used for the here described vaccine platforms is the forced exchange of S2[VP2] and S6[VP5] encoding serotype immunodominant outer shell proteins. In addition, reverse genetics opened possibilities to manipulate viral functions by genetic modification of BTV in order to develop replicating vaccine platforms (MLV platforms).

##### “Serotyped” Live-Attenuated Vaccines

A new generation of experimental LAVs is based on LAV serotype 6 (BTV6/net08) (35, 147) with exchanged outer shell proteins (Figure 3E). This LAV platform has been studied for serotypes 1 and 8 and results in nonvirulent so-named “serotyped” LAV1 and 8, respectively (148). Vaccination with monovalent or a trivalent cocktail of serotyped LAVs protects sheep against virulent BTV and induces serotype specific neutralizing antibodies against included serotypes. To combat multiple serotypes, tailor-made cocktails of serotyped LAVs could be freely applied, since reversion to virulence by reassortment between serotyped LAVs will be negligible because of the common LAV backbone. Consequently, these LAVs share most genome segments and the risk of arise of virulent variants has minimized. Further, negative interference of protection by different serotyped LAVs will be minimized because of the shared replication machinery. Though, reversion to virulence of serotyped LAVs by point mutations is a potential risk (149). Furthermore, elevated body temperature, clinical signs and viremia have been observed after vaccination (148). Therefore, safety of serotyped LAVs is incomplete and debatable as viremia could lead to undesired onward spread of vaccine virus by midges. Altogether, cocktails of serotyped LAVs are safer than cocktails of conventional LAVs, but their safety is still debatable due to the risk of reassortment events with wtBTV.

##### Disabled Infectious Single cycle (DISC) vaccines

Reverse genetics has initiated the development of improved vaccines by genetic modification of BTV. The Disabled Infectious Single Cycle (DISC) vaccine platform is based on BTV1 without expression of essential viral helicase VP6 (150). DISC vaccine virus cannot fulfill the virus replication cycle by lack of *de novo* VP6 synthesis, and DISC vaccine viruses must be produced by *in trans* complementation in cells expressing VP6 protein (Figure 3F). Consequently, DISC vaccine virus infects cells of the vaccinated ruminant only once, since infectious BTV cannot be assembled. The abortive replication of DISC vaccine virus induce a full blown immune response closely mimicking BTV infection, and results in mRNA synthesis and expression of all BTV proteins, except for VP6. The DISC vaccine platform has been applied for several serotypes by exchange of the serotype specific outer shell. Monovalent DISC vaccine and some DISC cocktail vaccines have been studied in sheep and cattle (150–152). A single DISC vaccination is protective in both sheep and cattle. In these studies, DISC vaccination contains crude cell lysate with ±1 × 10^7^ TCID_50_/ml per DISC vaccine virus, but the minimal protective dose of DISC vaccine has not been determined yet. DISC vaccine virus is completely safe with respect to clinical signs and viremia, although transient positivity by PCR has been observed short after vaccination. Monovalent DISC vaccine for serotype 8 protects sheep against clinical signs and viremia (150). Trivalent DISC vaccine for serotype 2, 4, and 8 completely protects sheep and cattle at 3 weeks post booster vaccination against virulent BTV2, 4 or 8 (151). Hexavalent DISC vaccine for serotypes 1, 2, 4, 8, 13, and 21 also protects against virulent BTV2 or 8 (152). Moreover, hexavalent DISC vaccine induced neutralizing antibodies against all included serotypes after booster vaccination, suggesting protection for all these serotypes. The deletion in S9[VP6/NS4] of DISC vaccine abolishes expression of VP6 but also of recently discovered NS4 protein. NS4 protein is not essential for virus replication *in vitro* but antagonizes Interferon-I expression *in vivo* (26, 153). Likely, lack of NS4 will positively affect the immune response by DISC vaccination, although this has not been studied. So far, the studied DISC vaccine consists of crude cell lysate with minor amounts of complemented VP6 protein. The DIVA potential of the DISC vaccine platform based on VP6 or NS4 has not been investigated yet.

##### Disabled infectious single animal (DISA) vaccines

**Figure 4:**
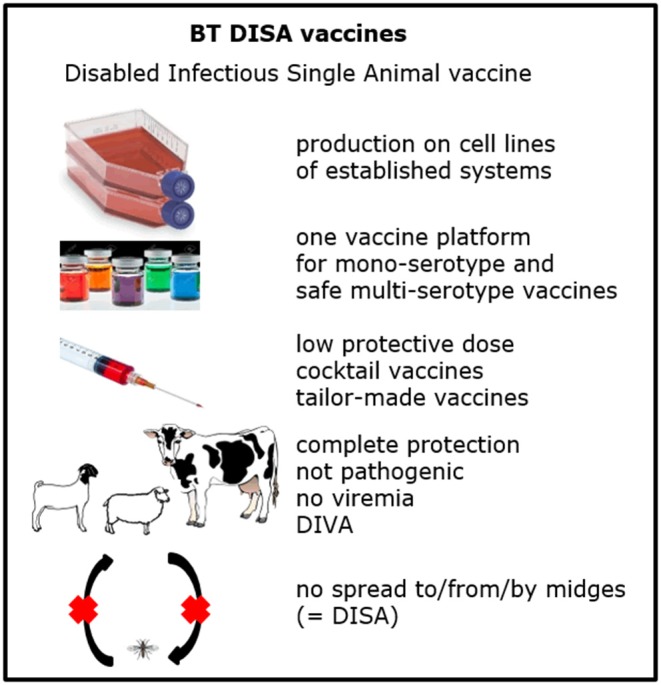
Overview of the vaccine profile of the DISA vaccine platform.

The principle of Disabled Infectious Single Animal (DISA) is a blockade on transmission of vaccine virus by midges. The key of DISA vaccine platform is knockout of NS3/NS3a protein by a deletion in S10[NS3/NS3a] (Figure 3G). Both NS3 and NS3a protein are not essential for virus replication *in vitro*, whereas virus release from *Culicoides* cells depends on NS3/NS3a protein (27). DISA vaccine virus cannot propagate in competent midges after intrathoracic inoculation (154). Moreover, a small in-frame deletion of 72 amino acid codons in NS3/NS3a protein leads to the same phenotype (155). Furthermore, DISA vaccine virus cannot pass the midge midgut barrier after blood feeding, and cannot reach the salivary glands, and therefore will not be secreted in saliva (155). It has been proposed that DISA vaccine virus only replicates near the vaccination site (156). Altogether, onward transmission of DISA vaccine has been blocked on uptake as well as on secretion (Figure 4). The DISA vaccine platform has applied for several serotypes by single S2[VP2] exchange (157), by exchange of both outer shell proteins as described (128, 148), and by incorporation of chimeric S2[VP2] of serotype 1 and 16 (157). DISA vaccine can be produced in established vaccine production facilities similar as for production of LAV or BTV antigen.

Virulent BTV8 without NS3/NS3a expression does not cause disease in sheep, indicating that NS3/NS3a is essential for virulence (156). Several deletions in S10[NS3/NS3a] are genetically unstable, but NS3/NS3a expression and pathogenicity of BTV has never been restored (158). Replication of DISA vaccine virus is required for protection but does not cause viremia (156). DISA vaccine based on BTV6/net08 (159) is superior to that based on a BTV1 or BTV8/net06 backbone with respect to protection, and completely protects sheep against virulent BTV8 at 3 week post single vaccination (156). Prime-boost DISA vaccination results in lasting serotype specific protection (160). A standardized dose of 2 x 1 ml 10^5^ TCID_50_/ml DISA vaccine was subcutaneously administered in these studies, however, a 100 times diluted vaccine dose, 2 × 1 ml 10^3^ TCID_50_/ml, and intramuscular or intravenous vaccination with a standard dose all results in VP7 seroconversion (161). Recent vaccination-challenge studies demonstrate early and serotype specific protection after intramuscular vaccination of cattle with DISA vaccine with the small in-frame deletion (van Rijn et al. personal communication). Further, prime-boost intramuscular vaccination of sheep with a pentavalent cocktail of DISA vaccines for the “European” serotypes 1, 2, 3, 4, and 8 based on the same DISA platform protects against virulent BTV2 or 8, suggesting that sheep are protected for all five serotypes (van Rijn et al. personal communication) (Figure 4). Lack of NS3/NS3a protein likely enhances the interferon mediated immune response, since NS3/NS3a counteracts the innate immune response, and in particular the type I interferon (IFN-α/β) pathway by different mechanisms (162–164).

Finally, DISA vaccine is DIVA compatible with panBTV PCR tests targeting S10 (36, 165–167), since the deletion in S10 partially overlaps their PCR targets (168). Furthermore, BTV infection induces NS3 Abs (102), and DISA vaccine is therefore DIVA compatible with an experimental NS3 competitive ELISA (103). Indeed, the NS3 competitive ELISA differentiates BTV infected from DISA vaccinated animals (156, 160) (Figure 4). Studies in large animal groups, preferably in the field, are required to determine the final vaccine profile of these DISA vaccines. Although DISA vaccines are scientifically safe and rationally acceptable, the current hurdle is permission to perform field trials with DISA vaccines as these BTVs with a small deletion are classified as GMOs.

##### NS4 knockout vaccines

BTV without NS4 expression from S9[VP6/NS4] could be an attractive vaccine platform, as NS4 is a determinant of virus virulence (153) (Figure 3G). Three silent point mutations in the VP6 open reading frame result in a mutated NS4 start codon and two in-frame stop codons in the open reading frame of NS4 adjacent downstream the NS4 start codon and selectively abolish NS4 expression. The BTV NS4 knockout mutant did not induce elevated body temperature nor clinical signs in sheep, while neutralizing antibodies were raised against the BTV NS4 knockout mutant similar as by wtBTV infection. Unfortunately, viremia was observed after inoculation and lasted for up to 28 days and protection against BTV challenge was not studied. Recovery of NS4 expression, and thus virulence, is minimized by the triple point mutation. However, due to its lasting viremia, and potential onward spread by midges, reversion to virulence cannot be excluded. BTV NS4 knockout mutants are not further explored as potential vaccine yet, but a NS4 knockout mutant of the related African horse sickness virus has shown promising results in horses (169). The BTV NS4 knockout mutant replicates in cell lines as used for BTV propagation, indicating that production of BT NS4 knockout vaccines should be possible in established facilities. Similar to other published BT vaccine platforms, this platform will be applicable for many serotypes by exchange of serotype specific outer shell proteins.

The here described MLV platforms are based on one appropriate virus backbone used to vary one or two segments encoding serotype specific outer shell proteins. Thus, each platform share 8 or 9 out of 10 genome segments including one mutated segment for most of the vaccine platforms (Figures 3E–G). Consequently, the vaccinated animal induces humoral as well as T-cell mediated responses directed against all BTV proteins, except for the one encoded by the modified genome segment. The lack of expression leads to attenuation (NS4 knockout platform), abortive replication (DISC platform), or a combined non-transmissibility, non-virulence and DIVA (DISA platform). Importantly, the shared backbone prevents reversion of virulence by reassortment between vaccine viruses. Tailor-made cocktail vaccines or foreseen broad protective vaccines are equally safe as single vaccines. Nonetheless, these modern vaccine platforms based on reverse genetics are genetically modified organisms (GMOs), and more efforts must be invested to proof their complete safety but eventually could allow a lower biosecurity level for vaccine production.

Expectedly, the here described approaches could be combined by improved technologies in the future. Inactivated DISA vaccine combines DIVA and avoids the GMO issue. Reverse genetics for circulating or (re-)emerging wtBTVs will be quickly developed in the future. In combination with the modification according to the described MLV platforms will result in a safe and protective vaccine that will induce an immune response exactly matching to the field BTV strain. More importantly, this strategy will avoid arise of virulent variants by reassortment events between vaccine strain and wtBTV.

## Concluding Remarks

The main vaccine standards are efficacy, safety, affordability, DIVA, and acceptance by the community. Marketed LAVs and inactivated BT vaccines are both successful to control BT outbreaks but have their specific pros and cons. LAVs are cheap but considered unsafe, while inactivated BT vaccines are safe but more expensive (Table 1).

**Table 1:** Evaluation of vaccine profiles of marketed and experimental vaccines.

**Marketed vaccines**	**Protection**	**Safe**	**Affordable**	**Acceptable**	**DIVA**	**Main advantages**	**Main disadvantages**	**Remarks**
Live-attenuated vaccine (LAV)	Yes	No^1^	Yes	Yes/No^2^	No	Cheap full blown response	No DIVA unsafe, in particular for cocktails	^1^: Virulence (residual and/or reversion) ^2^: Licensed in African countries, not accepted in other countries
Inactivated vaccine	Yes^1^	Yes	Yes^2^	Yes^3^	No	Safe	No DIVA expensive	^1^: Most require annual revaccination ^2^ : More expensive than LAVs ^3^: Available for limited serotypes
**Experimental vaccines**	**Protection**	**Safe**	**Affordable**	**Acceptable**	**DIVA**	**Main advantages**	**Main disadvantages**	**Remarks**
**PROTEIN VACCINES**								
VP2 subunit vaccine	Yes^1^	Yes	No^2^	Yes	Yes^3^	Commercial DIVA test	Expensive late onset	^1^: Requires booster vaccination ^2^: Likely expensive ^3^: Commercial VP7 cELISA
VLP vaccine	Yes^1^	Yes	No^2^	Yes	Yes^3^	Safe	Expensive late onset	^1^: Requires booster vaccination ^2^: Likely expensive ^3^: Experimental NS ELISAs
“Serotyped” inactivated vaccine	Yes^1^	Yes	Yes^2^	Yes	No	Traditional vaccine production	No DIVA	^1^: Requires annual revaccination ^2^: More expensive than LAVs
**VIRAL VECTOR VACCINES**								
VP2 expressing vector vaccine	Yes^1^	Yes	?^2^	Yes^3^	Yes^4^	Commercial DIVA	Late onset unknown efficacy in ruminants	^1^: Requires booster vaccination ^2^: Not or marginally tested in ruminants ^3^: Safe viral vector but GMO ^4^: Commercial VP7 cELISA
NS expressing vector vaccine	Yes^1^	Yes	?^2^	Yes^3^	Yes^4^	Commercial DIVA test proposed broad protection	Late onset unknown efficacy in ruminants	^1^:Requires booster vaccination ^2^: Not or marginally tested in ruminants ^3^: Safe viral vector but GMO ^4^: Commercial VP7 cELISA
**REPLICATING VACCINES (MLV)**								
“Serotyped” LAV	Yes	No^1^	Yes	Yes^2^	No	Traditional vaccine production full blown response	No DIVA viremia	^1^ : Viremia suggests onward transmission cocktails will be safer than of LAVs ^2^: BTV reassortant (GMO issue)
DISC vaccine	Yes	Yes^1^	Yes^2^	Yes^3^	No	Abortive vaccine replication combined with full blown response	No DIVA high dose	^1^: Abortive replication ^2^: Likely high protective dose ^3^: Complemented BTV (GMO issue)
DISA vaccine	Yes	Yes^1^	Yes	Yes^2^	Yes^3^	No vaccine transmission combined with DIVA and full blown response	GMO	^1^: Not transmittable by midges ^2^: Deletion BTV (GMO issue) ^3^: Experimental NS3 cELISA
NS4 knockout vaccine	Not tested	No^1^	Yes	Yes^2^	No	Cheap	No DIVA efficacy unknown	^1^: Viremia suggests onward transmission ^2^: BTV knockout mutant (GMO issue)

*For each vaccine, comments on some main vaccine standards (numbered) are described in the column “Remarks”. In addition, the most important advantages and disadvantages of each of the vaccines are indicated based on the current available data*.

In addition, both marketed BT vaccines lack DIVA and have limitations with regard to safely combat multi-serotype situations in the field. Experimental BT vaccines, such as protein vaccines, viral vector vaccines, and replicating vaccines, have been developed and some are well studied but none have been licensed yet. Nonetheless, new vaccine candidates show improvement for one or more of the vaccine standards. However, their final vaccine profile has not been definitely determined yet, although some can be assumed based on the present data. Because of this incompleteness, comparison of their final (expected) vaccine profiles is hardly possible (Table 1).

### Efficacy

MLVs are likely more effective than BTV protein vaccines and viral vector vaccines, since replicating BT vaccines can induce humoral and T-cell mediated immune responses against almost every BTV protein, and show protection after single vaccination. Further, broad protection is likely easier to achieve, since more conserved epitopes among BTVs as well as serotype specific epitopes are exposed to the immune system. Furthermore, application for multiple serotypes have been successfully studied for several MLV vaccine platforms.

### Safety

BTV protein vaccines and viral vector vaccines are completely safe due by the absence of infectious BTV, although local reactions on the vaccination site could be induced depending on the used adjuvant. Safety of MLVs varies between different platforms. DISC and DISA vaccines do not cause viremia or adverse effects, and are blocked on spread of vaccine virus between animals. “Serotyped” LAVs and NS4 knockout vaccine are not 100% safe, since a significant viremia could lead to onward transmission of vaccine virus by midges and might transmit vertically to the fetus.

### Affordability

The price per dose as well as per protected animal is hard to calculate for these experimental vaccines. Expectedly, vector vaccines and MLVs will be cheaper than BTV protein vaccines, since replication of MLVs in the receipt will trigger the immune system better than BTV protein based vaccines. Generally, the protective dose will be lower for replicating vaccines. Eventually, affordability will depend on vaccine efficacy but also on required boost vaccinations.

### DIVA

BTV protein vaccines and viral vector vaccines are DIVA compatible with the commercially available and widely used VP7 ELISA if VP7 protein is not part of the vaccine. Therefore, DIVA monitoring will be very easy and cheap by testing bulk milk samples, in particular if combined with other monitoring programs like for Infectious Bovine Rhinotracheitis and Bovine Viral Diarrhea (170, 171). This will support eradication programs in an affordable manner, and will increase the acceptance of DIVA testing. DISA vaccination can have the same advantage, since an experimental NS3 ELISA accompanying the DISA vaccine platform has been developed but this ELISA is not extensively validated and evaluated for milk samples yet.

### Acceptance

BTV protein vaccines will be acceptable, since these are completely safe. Even more, unnoticed pathogens, like in contaminated serum used for antigen production, will be inactivated or removed during down processing of antigen. With regard to viral vector vaccines and MLVs, control of used components in advance as well as of produced vaccine batches is extremely important. Many incidences of contaminated batches of replicating vaccines have been reported (172). Complete synthetic culture medium will avoid this disadvantage of replicating vaccines but is still quite expensive. Nevertheless, all here described MLV vaccine platforms are classified as GMOs, and licensing and acceptance will be costly due to extra safety trials. DISC and DISA platforms are based on disabled BTV due to a single deletion, and their safety has been scientifically predicted and has been proven in many sheep and cattle trials.

Several research groups have developed experimental BT vaccines and BT vaccine platforms showing promising vaccine profiles close to animal trial required for official vaccine registration. Licensing and launching next-generation BT vaccine, however, will mainly depend on the need for better than current vaccines in order to combat Bluetongue in mono-serotype situations to eradicate the disease and in multi-serotype endemic situations to minimize economic losses.

## Author Contributions

The author confirms being the sole contributor of this work and has approved it for publication.

### Conflict of Interest

The author declares that the research was conducted in the absence of any commercial or financial relationships that could be construed as a potential conflict of interest.
